# Survey on Use of Local and Systemic Corticosteroids in the Management of Chronic Rhinosinusitis with Nasal Polyps: Identification of Unmet Clinical Needs

**DOI:** 10.3390/jpm12060897

**Published:** 2022-05-29

**Authors:** Eugenio De Corso, Carlotta Pipolo, Elena Cantone, Giancarlo Ottaviano, Stefania Gallo, Frank Rikki Mauritz Canevari, Alberto Macchi, Giulia Monti, Carlo Cavaliere, Ignazio La Mantia, Sara Torretta, Francesco Bussu, Emanuele Scarano, Paolo Petrone, Angelo Ghidini, Daniela Lucidi, Massimiliano Garzaro, Matteo Trimarchi, Veronica Seccia, Giulio Cesare Passali, Daria Salsi, Domenico Cuda, Ernesto Pasquini, Luca Malvezzi, Stefano Settimi, Gaetano Paludetti, Jacopo Galli

**Affiliations:** 1 Otorhinolaryngology Unit, Head and Neck Department, Fondazione Policlinico Universitario A. Gemelli Hospital IRCCS, 00168 Rome, Italy; 2 Otorhinolaryngology Unit, Head and Neck Department, ASST Santi Paolo e Carlo Hospital, 20142 Milan, Italy; 3 Neurosciences, Reproductive and Odontostomatologic Sciences, Unit of Ear, Nose and Throat, Federico II University, 80131 Naples, Italy; 4 Head and Neck Department, Otorhinolaryngology Federico II, Unit of Ear, Nose and Throat, 80131 Naples, Italy; 5 Department of Neurosciences, Section of Otolaryngology, University of Padua, 35128 Padua, Italy; giancarlo.ottaviano@unipd.it; 6 Clinica Otorinolaringoiatrica, Ospedale di Circolo e Fondazione Macchi, ASST Sette Laghi, 21100 Varese, Italy; stefania.gallo@me.com; 7 UOC di Otorinolaringoiatria IRCCS Policlinico San Martino Genova, Dipartimento DISC Università di Genova, 16132 Genova, Italy; canevari@edu.unige.it; 8 Clinica di Otorinolaringoiatria Asst-Settelaghi, Upload Center, Università degli Studi dell’Insubria, 21100 Varese, Italy; macchi.doc@gmail.com (A.M.); giuliamonti2403@gmail.com (G.M.); 9 Department of Sense Organs, Sapienza University of Rome, 00185 Rome, Italy; carlocavaliere1985@gmail.com; 10 UOC di Otorinolaringoiatria, Università di Catania, 95123 Catania, Italy; igolama@gmail.com; 11 Fondazione IRCCS Ca’ Granda Ospedale Maggiore Policlinico, 20122 Milan, Italy; sara.torretta@gmail.com; 12 Department of Clinical Sciences and Community Health, University of Milan, 20122 Milan, Italy; 13 Otolaryngology Division, University of Sassari, 07100 Sassari, Italy; fbussu@uniss.it; 14 Azienda Ospedaliera Cardinale Panico Tricase, 73039 Lecce, Italy; escarano@tim.it; 15 Directorate General, ASL BA, 70123 Bari, Italy; info@paolopetrone.it; 16 S.C. di Otorinolaringoiatria Azienda USL di Reggio Emilia/IRCCS, 42122 Reggio Emilia, Italy; ghidini.angelo@ausl.re.it; 17 Department of Otolaryngology—Head and Neck Surgery, University Hospital of Modena, 41121 Modena, Italy; dani.lucidi@gmail.com; 18 ENT Division, Ospedale Maggiore della Carità di, University of Eastern Piedmont, 13100 Vercelli, Italy; massimiliano.garzaro@uniupo.it; 19 Division of Head and Neck Department, Otorhinolaryngology Unit, IRCCS San Raffaele Scientific Institute, 20132 Milan, Italy; trimarchi.matteo@hsr.it; 20 Otolaryngology, Audiology, and Phoniatric Operative Unit, Department of Surgical, Medical, Molecular Pathology, and Critical Care Medicine, Pisa University Hospital, 56124 Pisa, Italy; veronicaseccia@gmail.com; 21 Fondazione Policlinico Universitario A. Gemelli IRCCS, Università Cattolica del Sacro Cuore, 00168 Rome, Italy; giuliocesare.passali@unicatt.it; 22 U.O.C. Otorinolaringoiatria Azienda USL di Piacenza, 29121 Piacenza, Italy; salsidaria@hotmail.com (D.S.); d.cuda@ausl.pc.it (D.C.); 23 Surgical Department, ENT Metropolitan Unit, Bellaria Hospital, 40139 Bologna, Italy; epasquini@yahoo.com; 24 Department of Otorhinolaryngology and Head and Neck Surgery, IRCCS Humanitas Research Hospital, 20089 Rozzano, Italy; luca.malvezzi@humanitas.it; 25 Department of Head-Neck and Sensory Organs, Catholic University of Sacred Heart, 00168 Rome, Italy; stefano.settimi@unicatt.it (S.S.); gaetano.paludetti@unicatt.it (G.P.); jacopo.galli@unicatt.it (J.G.)

**Keywords:** chronic rhinosinusitis, with nasal polyps, corticosteroids, survey, management, unmet needs

## Abstract

*Background*: Local and systemic corticosteroids have long been the workhorse in management of chronic rhinosinusitis with nasal polyps (CRSwNP), although there is no universally accepted modality of prescription. We carried out a survey in Italy to capture current trends in the use of topical and systemic corticosteroids in patients with CRSwNP. *Methods*: A survey was set up on Survey Monkey^®^. Each author distributed the link to the survey in an ad hoc manner and a total of 437 participants filled out the survey. *Results*: Mometasone furoate (79.3%) was the most frequently prescribed, administered daily by 61.9% of participants; the remaining preferred to discontinue treatment for brief periods to reduce side effects or to modulate the therapy in mild cases. The majority believe that a short cycle of systemic steroids should be prescribed for re-exacerbation of symptoms and that the number of cycles in the previous year should be evaluated to define control of the disease even if international guidelines do not provide clear indications on this topic. A certain degree of divergence emerged from responses regarding how long and the maximal dose of systemic steroids which place patients at high risk for adverse events. Finally, systemic corticosteroids seem to offer only temporary benefit on recovery of smell without guaranteeing long-term control even if the patient is adherent to topical corticosteroids. *Conclusions*: Our results highlight the need for clear guidelines on oral steroids, which could help supporting the use of a precision medicine approach, including indications for new biological agents.

## 1. Introduction

Chronic rhinosinusitis with nasal polyps (CRSwNP) is an inflammatory disorder [1] affecting about 4% of the population globally and with a significant impact on the quality of life (QoL) [2,3,4]. It is associated with substantial burden, not only for patients but also in terms of healthcare resources and loss of productivity, which is amplified in patients with poorly controlled disease [3]. Unfortunately, CRSwNP remains a challenge to manage given the difficulty in controlling symptoms and the frequent relapses [1,3], especially in a specific subset of patients with CRSwNP driven by a type 2 inflammatory response.

For several years, the standard management of CRSwNP consisted of thorough diagnostic work-up and medical therapy, together with meticulous follow-up, and, in selected cases, repeated surgeries [1]. Chronic inflammation is usually suppressed by steroids; therefore, local and systemic glucocorticoids have long been a workhorse in the management of CRSwNP [1]. For instance, intranasal steroid (INCS) sprays have become one of the most widely used strategies for long-term medical therapy. In fact, despite the highly variable pathophysiology of CRSwNP, both EPOS 2012 and EPOS 2020 recommended the use of INS, albeit with use that should be modulated depending on the severity of the disease [3,5]. Systemic steroids also have their place in disease management, and EPOS 2020 recommends their use, specifically to manage re-exacerbations and relapse by using brief cycles [5,6]. Unfortunately, a substantial portion of patients remain refractory even to maximal medical therapy. In addition, systemic steroids have well-known systemic adverse effects, especially at high doses and/or over prolonged periods, which is problematic when treating a chronic disease such as CRSwNP [6].

All these considerations highlight the need for tailored management of the individual patient and a precision-medicine-based approach [3,5] with the aim of limiting unnecessary and inefficacious treatment with systemic steroids. Unfortunately, there is no universally accepted modality of prescription of systemic steroids by clinicians, and indeed routine clinical practice is characterized by significant heterogeneity in terms of type, dosage, and treatment duration of systemic steroids.

Although clinicians use a wide array of modalities in prescribing corticosteroids, they all have experienced the unfortunate inability of systemic corticosteroids to reach long-term control of disease in a subgroup of patients, especially those with a type 2 inflammatory endotype [5]. Greater understanding of the molecular mechanisms of type 2 inflammation has led to the development of targeted biological drugs, in order to overcome these limitations [7,8,9]. Given the large variety of treatment options and guidance, management of CRSwNP is highly variable in routine clinical practice and often depends on the subjective clinical judgement of the clinician, as highlighted by previous studies [5,10,11,12,13,14]. In order to shed light on clinical practice in Italy, we carried out a nationwide survey to capture current trends regarding the use of topical and systemic steroids in patients with CRSwNP trying to highlight clinical unmet needs in this field.

## 2. Materials and Methods

### 2.1. Study Design

We conducted a survey on a representative sample of Italian Otorhinolaryngologists, with the aim of understanding the use of corticosteroids in the management of CRSwNP. With this purpose, a group of ENTs involved in the management of CRSwNP, and selected for their professional experience in rhinology, met remotely to discuss salient aspects in the management of CRSwNP with specific focus on unmet needs regarding the use of local and systemic steroids. Finally, a group of 437 ENT specialists (230 females and 207 males; mean age of 44 years) from all the regions of Italy were involved who worked at both University and Public Hospitals.

The authors were divided into five groups according to different topics: (i) use of local steroids in CRSwNP; (ii) use of systemic steroids in CRSwNP; (iii) definition of disease control based on steroid use; (iv) perioperative use of local and systemic steroids; (v) use of steroids in olfactory dysfunction associated with nasal polyposis. Each subgroup then suggested questions related to their defined survey theme based on a thorough review of the literature along with a comparison of routine management of CRSwNP. The questions were produced based on analysis of literature regarding the unmet needs in the use of corticosteroids in management of CRSwNP. A version of the questionnaire was firstly prepared by E.DC., E.C., and C.P., and after, submitted to the experts in the field that were involved as authors of this manuscript. All questions were then unformed for style and answer possibilities, in order to ensure a direct and standardized response that reflects the respondent’s experience. The experts provided a critical appraisal of all questions, refining a total of 38 final questions after an interactive two-step revision process. The final questionnaire is reported in Supplementary Material.

In order to be representative of Italian Otorhinolaryngologists’ experience, we performed a statistical sample size evaluation by the SurveyMonkey calculator using a 95% confidence interval (Z = 1.96) and with a margin of error of 5 units. The analyses showed that for our purpose, a sample size of at least 357 physicians was appropriate.

### 2.2. Survey Distribution

The survey was set up on Survey Monkey^®^. Each author distributed the link to the survey according to professional relationships (linking). The distribution modes used were not pre-established, but included professional contacts, working groups, local media, email, etc. The participants were also invited on the basis of geographical distribution so that the questionnaire could be circulated throughout the whole of Italy. No information about the participants was collected in order to keep the results of the questionnaire anonymous. No exclusion criteria for participation were applied, and all otolaryngologists managing patients with chronic rhinosinusitis were able to participate with no regard for academic or clinical role.

The questionnaire was distributed not only to otolaryngologists working in a hospital setting, but also to otolaryngologists with exclusive outpatient activity. The survey distribution started on 1 October 2021 and was closed on 31 December 2021. All the answers were considered appropriate and were included in the analysis. We performed a descriptive analysis and present some of the most significant results as histograms.

## 3. Results

Herewith, we report the results about the five main areas of interest of this manuscript. A total of 437 participants filled out the survey. Responses for each theme are described below.

### 3.1. Use of Local Steroids in CRSwNP

**Figure 1:**
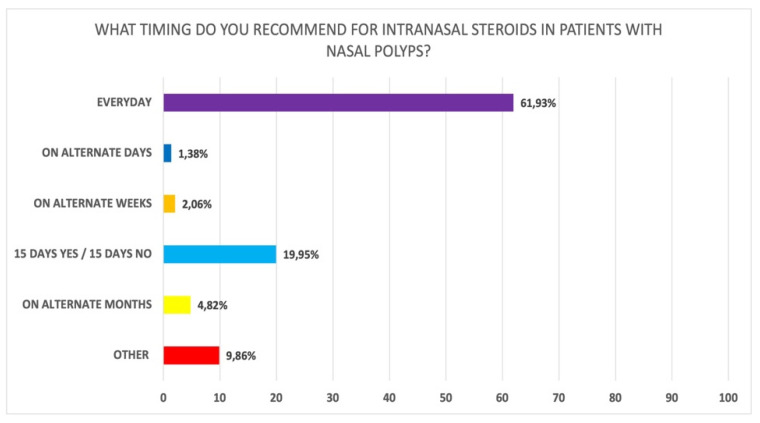
Recommended frequency of prescription of local corticosteroids in patients with CRSwNP (Q5 of Supplementary Materials File).

This area included 13 questions about steroid use, combination therapy, device, modality of administration, duration, posology, adherence, and adverse events. From the participants’ responses, mometasone furoate (79.3%) was the most frequently prescribed in the clinical practice, followed by budesonide (29.0%) (Q1 of Supplementary Materials). The most common modalities of administration were nasal spray (98.6%) and nasal douches (17.0%) (Q2 of Supplementary Materials). Local corticosteroids were frequently prescribed in association with nasal saline spray and saline irrigation (76.2%), but not necessarily in the same moment of the day (Q3 of Supplementary Materials). Regarding timing of prescriptions, local corticosteroids were administered daily by 61.9% of the ENTs involved in the survey (Q5 of Supplementary Materials; Figure 1); the remaining preferred to discontinue the treatment for brief periods to reduce side effects or modulated the therapy based on the severity of symptoms. Interestingly, 63.9% of participants fully agreed and 28.5% partially agreed that modulation of therapy should be applied in the routine clinical practice (Q9 of Supplementary Materials).

It is widely accepted, and supported by literature data, that long-lasting administration of topical corticosteroids is used to control chronic sinonasal inflammation. This aspect should be noted by the clinician during counselling at first evaluation of a CRSwNP patient. Nevertheless, follow-up is important to evaluate adherence, to modulate therapy, and to prevent adverse events. For this reason, we investigated the duration of the prescription of INCS at first evaluation and the time needed by the clinician to re-evaluate the patient. It was evident that local corticosteroids are usually prescribed for a long-term period before the next evaluation, and specifically for at least 3 (41.3%), 6 (12.61%), or 12 months (12.6%) (Q4 of Supplementary Materials). The majority of clinicians (73.9%) believed that adherence to treatment was quite variable, reporting an estimated 50–70% compliance of cases affected by CRSwNP. Adherence was measured by asking the patient if they have strictly followed the indication about daily number of administrations and duration of medication. In case of inconstant administration, the patient was considered as “non-adherent”. Although clinicians fully agreed (70.0%) or partially agreed (20.9%) that prolonged use of intranasal steroids has minimal local adverse effects (Q7 of Supplementary Materials) and no systemic side effects (78.9%) (Q10 of Supplementary Materials), they did, however, consider the potential for negative consequences. In particular, epistaxis (76.4%) and nasal dryness (70.4%) were the main negative consequences reported by ENTs involved in this survey (Q11 of Supplementary Materials). Interestingly, the clinicians held the opinion that some specific strategies may reduce the risk of local adverse events in real-life.

These include instructing the patient on correct application of the product (49.9%), how to aim the device (58.6%), and use of concomitant therapies (e.g., hyaluronic acid, emollients, etc.) (58.4%) (Q8–Q13 of Supplementary Materials; Table 1; Table 2). Finally, important contraindications for the use of intranasal steroids were bleeding risk (44.2%) and glaucoma (59.1%) for the possible increase in intraocular pressure (Q12 of Supplementary Materials).

### 3.2. Use of Systemic Steroids in CRSwNP

**Figure 2:**
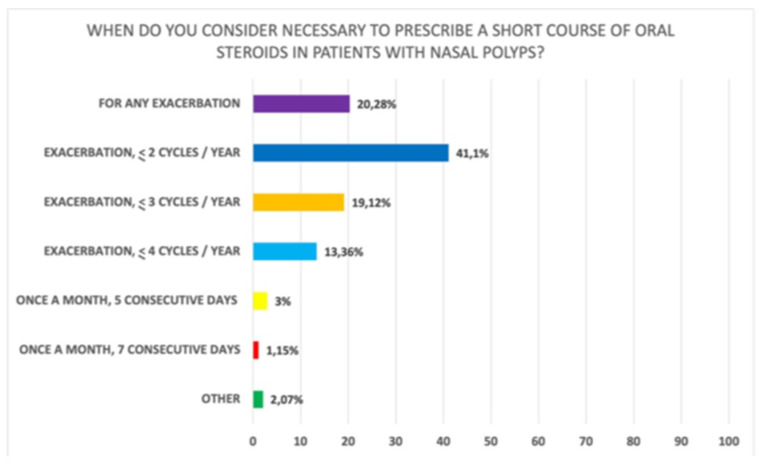
Perceived timing for prescription of a short course of oral corticosteroids in case of re-exacerbation of symptoms (Q17 of Supplementary Materials File).

Of note, 93.9% of clinicians believe that a short cycle of systemic steroids should be prescribed in case of re-exacerbation of symptoms. More specifically, 20.3% prescribe them regardless of the total number of cycles/year, 41.1% do not exceed two cycles/year, 19.12% do not exceed three cycles/year, and 13.36% do not exceed four cycles/year (Q17 of Supplementary Materials and Figure 2). The most widely used steroid is prednisone (45.7%), followed by deflazacort (38.8%) (Q16 of Supplementary Materials). With regard to the length of a short cycle, the majority of clinicians (89.8%) indicated 5–15 days, and out of these, 62.4% prescribed it for 5–10 days and 27.4% for 10–15 days (Q19 of Supplementary Materials). In addition, using prednisone as the reference, 47.1% prescribe 25 mg/day independent of body weight and only 31.4% adapt the dose to patients’ weight (Q18 of Supplementary Materials).

**Figure 3:**
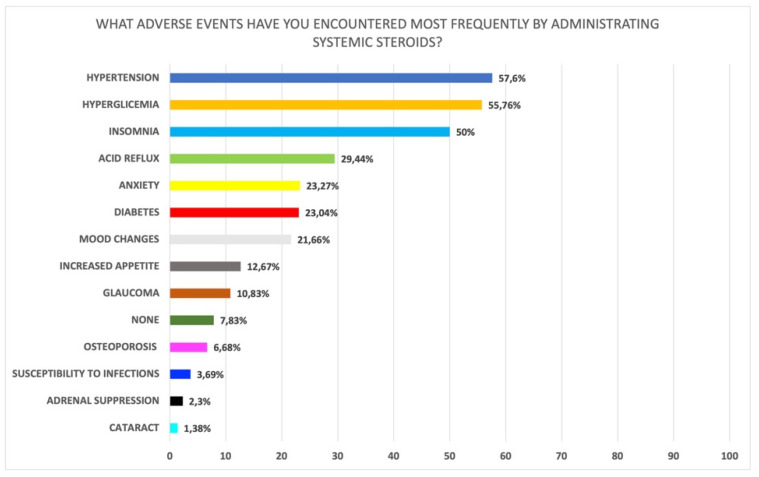
Adverse events most frequently observed with oral corticosteroids (Q26 of Supplementary Materials File).

Among the most commonly observed adverse events associated with systemic steroids (Figure 3), clinicians observed hypertension (57.6%), hyperglycemia (55.8%), and insomnia (50.0%). Gastroesophageal reflux (GERD) (29.5%), anxiety (23.27%), and other minor side effects were also observed as reported in Figure 3.

The otolaryngologists also discontinued systemic steroids due to adverse events, specifically for hypertension (54.0%), hyperglycemia/diabetes mellitus (43.2%), anxiety/depression/sleep disturbance (27.3%), gastritis/peptic ulcer (21.0%), and for other reasons. For all these reasons, clinicians stated that, if systemic corticosteroids are prescribed, patients should be closely followed for adverse events (28.5%) and that a systemic steroid with low mineralocorticoid activity should be used (17.8%) as well as other medical therapies (e.g., antihistamines and anti-leukotrienes) (18.8%) to reduce the need for steroids (Q25 of Supplementary Materials). In addition, they recommend monitoring blood pressure (91.0%) and blood glucose (87.5%) during treatment and referral to other specialists in case of complications (18.0%) (Q30 of Supplementary Materials).

The majority of clinicians (71.8%) reported that they do not administer depot systemic steroids (e.g., triamcinolone acetonide) in clinical practice for CRSwNP, with only 12.1% using depot systemic steroids in case of non-response to oral steroids (Q22 of Supplementary Materials). Surprisingly, only 54.7% of participants fully agreed with the recommendation not to use low-dose long-term steroids and 31.9% partially agreed (Q21 Supplementary Materials).

### 3.3. Definition of Disease Control Based on Steroid Use

Participants in this survey defined CRSwNP as severe uncontrolled as follows: 22.1% if symptoms persisted after two brief cycles of systemic steroids in the last year; 41.4% after three brief cycles of systemic steroids in the last year; and 32.4% after four brief cycles of systemic steroids in the last year (Q20 of Supplementary Materials). The heterogeneity of these responses appeared to be closely related to the lack of clarity of the guidelines on this topic. In fact, the ENTs partially agreed (56.8%) with the statement that international guidelines provide clear indications on the use of oral steroids for the treatment of CRSwNP. Only 19.0% fully agreed with this statement (Q23 of Supplementary Materials).

**Figure 4:**
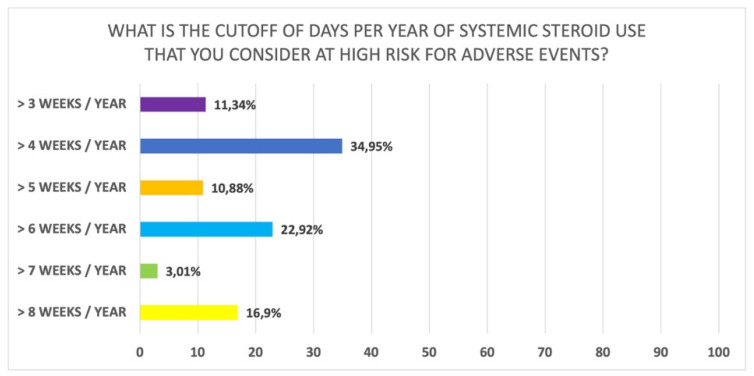
Number of days in a year considered at high risk of side effects (Q28 of Supplementary Materials File).

**Figure 5:**
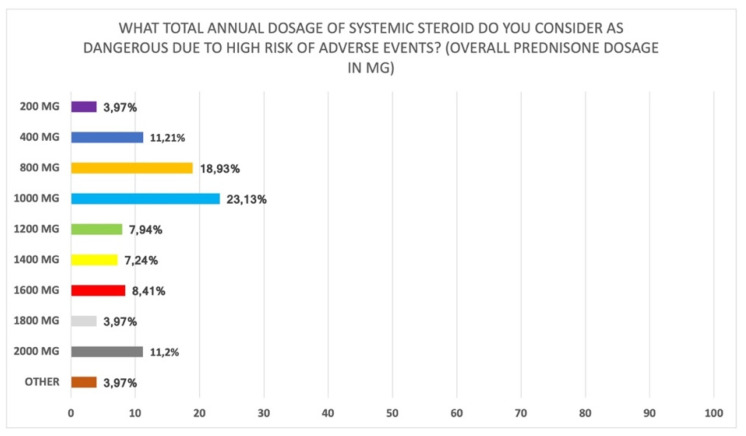
Total annual dose of corticosteroids considered at high risk of adverse events (Q29 of Supplementary Materials File).

Interestingly, most participants believe that the previous use of systemic steroids should be evaluated considering the number of cycles in the previous year (62.6%); 16.9% of participants also stated that the total number of days used in the last year should be considered, while 17.8% said that the total dose in last year should be taken into consideration (Q27 of Supplementary Materials). Furthermore, a large proportion of participants suggested that the amount of steroid used for asthma should also be considered [fully agree (68.2%); partially agree (25.3%)] (Q38 of Supplementary Materials). In addition, when asked about “how long per year do you consider systemic steroids to place patients at high risk for adverse events” there was heterogeneity in the responses: 35.0% stated 4 weeks per year, 22.9% more than 6 weeks per year, and 16.9% more than 8 weeks per year (Figure 4) (Q28 of Supplementary Materials). There was also heterogeneity of responses regarding the annual dose of systemic steroids which places patients at high risk for adverse events: 800 mg prednisone (18.9%), 1000 mg prednisone (23.1%), or 2000 mg prednisone (11.2%) (Q29 of Supplementary Materials) (Figure 5).

### 3.4. Perioperative Use of Local and Systemic Steroids

Regarding preoperatory management, based on the results of this survey, the majority (71.7%) of Italian ENTs do not discontinue intranasal steroids prior to surgical intervention (Q14 of Supplementary Materials). Furthermore, 66.7% normally prescribe a cycle of systemic steroids prior to surgical intervention, in order to reduce the load of inflammation and intraoperatory bleeding (24.7% always and 42.0% depending on severity of the disease). Regarding postoperative management, we assumed that all participants were aware that the use of topical corticosteroids is a long-lasting therapy, which is necessary to control sinonasal chronic inflammation and to prevent recurrence. Nevertheless, the timing for reintroducing them after surgery may be variable, and for this reason, we investigated this specific point. We observed that there was no complete agreement among participants, and specifically 29.0% reintroduce INCS after surgery as soon as mucosal healing of the entire nasal fossa is observed, 15.9% at 15 days after intervention, and 24.2% at 1 month after intervention (Q15 of Supplementary Materials).

### 3.5. Use of Steroids in Olfactory Dysfunction Associated with Polyposis

**Figure 6:**
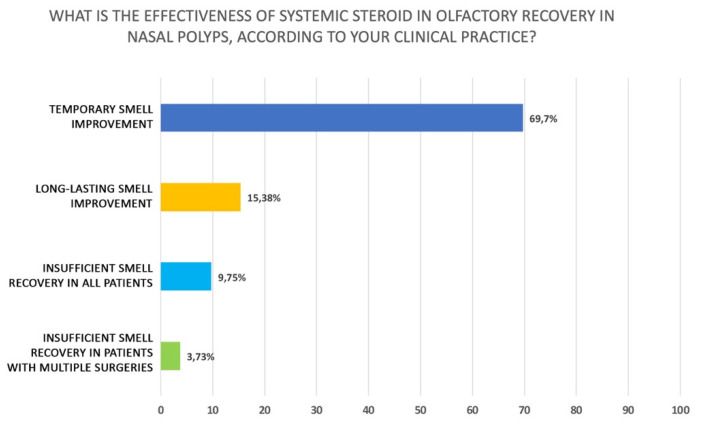
Perceived efficacy of oral corticosteroids in restoring olfaction (Q36 of Supplementary Materials File).

**Figure 7:**
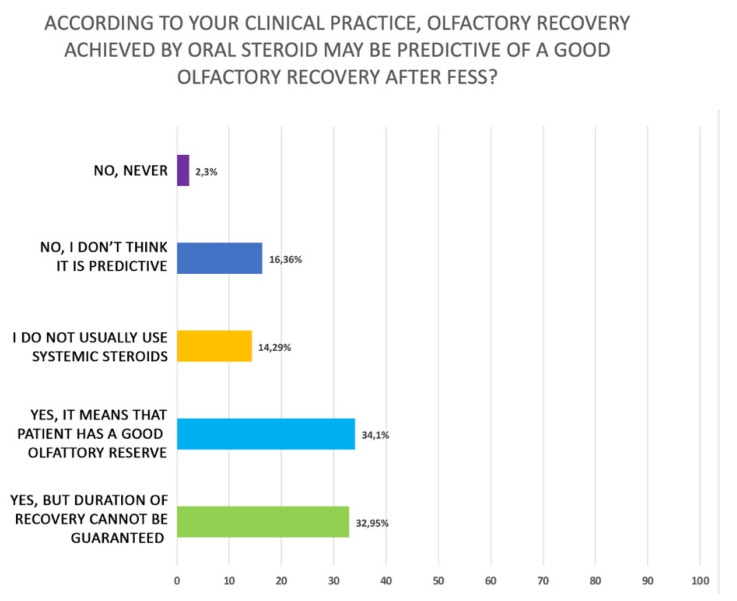
Perceived efficacy of oral corticosteroids on long-term control of olfaction (Q37 of Supplementary Materials File).

When olfactory impairment is the prevalent symptom in CRSwNP, the participants recommend a cycle of systemic steroids. Interestingly, half of them prescribe systemic corticosteroids only if the patient has already undergone therapy with an INCS (49.1%) (Q33 of Supplementary Materials). If a patient with CRSwNP complains mainly of olfactory disorders, the participants prescribing oral corticosteroids believe that a brief cycle for 7–15 days at full dose according to body weight should be prescribed (71.5%): specifically, 49.3% for 7 days and 22.2% for 15 days. The remaining participants consider that a short cycle of corticosteroids at low dose may also be an option (Q34 of Supplementary Materials). The clinicians did not prefer a particular agent in case of smell disorders. In fact, they use mainly prednisone (45.1%) and deflazacort (29.2%) (Q35 of Supplementary Materials), similarly to treatment for CRSwNP regardless of olfactory disfunction. Interestingly, 69.7% of participants stated that the benefit of systemic steroid therapy for recovery of olfactory function in CRSwNP is only transient, as shown in Figure 6 (Q36 of Supplementary Materials). Finally, only 34,1% of the clinicians that answered to this review disagreed about the statement that recovery of olfactory function with oral steroids is a good predictor of olfactory recovery after endoscopic sinus surgery and 32.95% declared that even if the recovery may be achieved after surgery, the duration cannot be guaranteed (Figure 7) (Q37 of Supplementary Materials).

## 4. Discussion

To the best of our knowledge, the present survey, involving a large number of Italian ENTs, is the only one assessing the use of local and systemic steroids in routine practice in patients with CRSwNP. One strength is the large number of participants which is likely to be representative of clinical ENT practice in Italy including around 6000 physicians. Several areas of agreement as well as lack of consistency in practice can be noted, highlighting unmet needs and calling for clarity in the management of this disease.

### 4.1. Use of Local Steroids in CRSwNP

Overall, considering the use of local steroids, a high degree of homogeneity in practice was seen. Mometasone furoate is the most frequently used intranasal steroid, and nasal sprays are without doubt the most preferred formulation [15]. Notwithstanding, factors such as the delivery method and prior sinus surgery may influence the efficacy of locally delivered steroids [16]. About half of ENT specialists recommend the combined use of an intranasal spray together with nasal saline irrigation. Interestingly, regarding timing of prescriptions, local corticosteroids were administered daily by at least 60% of the ENTs involved in the survey and the majority prescribe an intranasal long-term steroid treatment until the next visit. The majority of interviewed ENT did not interrupt local corticosteroids before surgery (71.66%) and restart it as soon as possible after surgery, possibly within one month. The authors of this review believe that it is very important to motivate patients towards long-term use of local corticosteroids even after surgery to reduce the risk of recurrence [10,17].

The perception appears that most patients are adherent to daily intranasal steroids, and most felt that these preparations are not associated with significant systemic adverse events. Nevertheless, the remaining clinicians commonly prescribe brief weekly or monthly intervals in the attempt to minimize side effects. It is clear that the perceptions of clinicians are that local corticosteroids should be prescribed daily to be effective, but that the fear of side effects or loss of adherence to treatment may influence the timing of prescription. Some clinicians also feel that it is important to advise patients on the correct administration of INCS. In agreement with EPOS guidelines, most prescribers tend to modulate the use of INCS based on severity of presentation, modifying the daily dose regimen with the possibility for brief periods of discontinuation [5]. Thus, many clinicians routinely administer intranasal corticosteroids to patients with CRSwNP according to precision medicine and therefore based on the severity of the disease. Epistaxis and nasal dryness were the most frequently encountered local adverse events, which could be reduced through correct application, discontinuation for a brief period, and use of emollients. Interestingly, it has been observed that while side effects are common with intranasal sprays, these may be related to incorrect application technique rather than to the medication itself [18]. This emphasizes the need to instruct patients on the proper application technique, which is routinely performed by roughly half of the participants in this survey. This is relevant since side effects may also reduce compliance to therapy [18,19]. Moreover, the correct use of topical steroids guarantees higher efficacy of the drug, which reaches the nasal polyps instead of the nasal septum or pharynx. Overall, perceived adherence to INCS (70%) appears to be in line with other studies [20,21,22], highlighting the need to increase awareness of difficulties in adherence during the physician–patient interview. It is also clear from results of this survey that the costs of local corticosteroids over the long term may influence adherence.

The panel of authors coordinating this survey agreed that topical nasal corticosteroids should be prescribed daily in CRSwNP. Nevertheless, the results of this survey clearly showed that fear of side effects may influence appropriate prescription. The need to instruct patients on proper application technique in order to limit side effects and improve adherence to therapy should be emphasized as noted by other authors [23]. In case of side effects despite due precautions, a change in modality of administration—or adjuvant—action should be taken. A small proportion of ENT interviewees declared that in case of side effects such as dryness and bleeding or if the patient is intolerant to the use of nasal spray, they propose a nasal douche with diluted local corticosteroids. If, finally, side effects persist, discontinuation of treatment should be considered [24].

### 4.2. Use of Systemic Steroids in CRSwNP

There was little consensus on the use of systemic steroids in routine practice, although prednisone and deflazacort were the most widely prescribed agents. Most clinicians prescribe a systemic steroid in cases of recurrence but without exceeding 2–3 cycles per year, although 20% prescribe oral steroids whenever a recurrence is seen. Additionally, while almost half prescribe 25 mg/day prednisone independently of body weight, around 30% consider body weight when prescribing. However, consensus on therapy duration is quite high with a vast majority prescribing an oral steroid in a range from 5 to 15 days.

The lack of homogeneity in practice might be partially explained by the lack of specific guidance in terms of duration and dose for systemic steroids in current guidelines [5]. Indeed, the majority of participants stated that international recommendations regarding the use of oral steroids for CRSwNP are not adequate. Moreover, the current evidence for the use of systemic steroids in patients with CRSwNP is considered to be of low quality [15,25,26]. Despite the use of systemic corticosteroids, a low dose in the long term is not recommended for the treatment of CRSwNP as stated by EPOS 2020 [5]. Surprisingly, only 54.7% of participants fully agreed with this recommendation and 31.9% partially agreed. This indicates a possible gap for harmonization of current practice, i.e., the need for additional education of prescribers. There was also little agreement on how to evaluate the use of systemic steroids with either number of annual cycles, total annual dose, or number of days.

As expected, the most frequently observed adverse events with systemic steroids were hyperglycemia, gastric reflux, insomnia, hypertension, and diabetes [6]. While on one hand, the majority of prescribers routinely recommend monitoring blood pressure and glycemia; on the other, there was little unanimity on how to manage recurrent symptoms using systemic corticosteroids in patients with comorbidities (cardiac disease, hypertension, diabetes, etc.) that place them at higher risk during oral steroid use; ENTs prefer to avoid systemic steroids and try to monitor side effects, while looking for alternative therapies and use of steroids with low mineralocorticoid activity. Interestingly, it was believed that fewer patients are adherent to oral steroids compared to intranasal steroids. Indeed, the majority of ENTs stated that the adherence is over 77% (Q32). Finally, the risk that patients begin to abuse corticosteroids should be taken into account, which may also increase the risk of important side effects.

### 4.3. Definition of Disease Control Based on Steroid Use

There was little congruence on how to define severe uncontrolled CRSwNP: responses varied from uncontrolled disease after two brief cycles of systemic steroid to more than four cycles in the last year. These responses reflect differences in current guidelines, and further highlight that there is a lack of clear guidance on the use of steroids. However, the majority agrees or mostly agrees that long-term low-dose oral steroids, as well as depot systemic steroids, should be avoided, in agreement with the literature. There also seemed to be agreement that use of steroids for asthma should also be considered when evaluating steroid use in CRSwNP, leading to a higher incidence of adverse effects when multiple steroid courses are administered, and supporting a multidisciplinary approach in these patients.

The data concerning the use of systemic corticosteroids are particularly heterogeneous. The authors of this manuscript believe that it is necessary to underline that, in accordance with international guidelines, a systemic corticosteroid should be used only for the exacerbation of symptoms and that 2–3 cycles per year should not be exceeded [3,6,27,28]. However, the results of this survey highlight the need for consensus about the limit to consider administration of systemic steroids as maximal medical therapy. Precise indications should be reached in the future and shared with the ENT community in order to clarify when a shift to targeted therapy with biologics is indicated.

### 4.4. Perioperative Use of Local and Systemic Steroids

Most prescribers did not feel the need to discontinue intranasal steroids prior to functional endoscopic sinus surgery (FESS), although one-quarter discontinue at least one week before. There was also a lack of agreement on when an intranasal steroid should be restarted following FESS or on systemic steroids prior to intervention. As about the use of oral steroids, this indicates that there is a need for clear recommendations on these aspects in current guidelines even though different reviews state the efficacy in improving perioperative bleeding, operative time, and reduction in hospital stay in patients who received oral steroids [6]. The authors of the present study believe that local corticosteroids should not be discontinued before FESS and resumed as soon as possible after surgery. Systemic corticosteroids prior to surgery should be considered as they may be effective in improving perioperative bleeding, operative time, and reduction in hospital stay [5].

### 4.5. Use of Steroids in Olfactive Dysfunction Associated with Polyposis

Both intranasal and systemic steroids appear to have a benefit on olfactory dysfunction, with systemic treatment being the most effective [29,30]. In the case that olfactive disorders are the prevalent symptom in CRSwNP, about half prescribe an oral steroid only if the patient has undergone a prior trial with an intranasal steroid, while one-quarter referred that they always prescribe a systemic steroid. However, almost all prescribe an oral steroid for olfactory disorders for 15 days or less at the full dose according to body weight. Most observe only transient improvement in olfactory function after prescribing an oral steroid, and most also felt that olfactory function with oral steroids could be a good predictor of olfactory recovery after FESS, although the reasons varied. Regarding olfaction, the results of the survey are clear in highlighting that systemic corticosteroids can offer only temporary benefit on recovery of smell without ensuring long-term control even if the patient is adherent to topical corticosteroids. This limitation becomes particularly important in those patients in whom olfactory dysfunction is predominant and for whom therapeutic alternatives are strictly necessary. The authors believe that in the management of sense of smell in CRSwNP patients, there is an urgent unmet need for new therapies that can improve this outcome quickly and sustainably over time. The results obtained in trials and in real-life settings with biologics seem to be very encouraging and may represent an important step to change the management approach to olfactory disorders associated with CRSwNP [27].

## 5. Conclusions

The results of the present survey highlight several interesting aspects on the use of topical and systemic corticosteroids in the treatment of CRSwNP. More homogeneous responses were obtained with respect to the use of topical corticosteroids, while a certain degree of divergence emerged from the responses on the use of systemic steroids. On the one hand, most clinicians appear to be aware of the side effects of corticosteroids; however, on the other, the responses of participants were very heterogeneous, especially with regard to the use of systemic corticosteroids and number of annual cycles, total annual dose, and number of days per cycle. The different methods of administration may depend on various factors. Systemic steroids are considered to be very effective even if not for long-term treatment, but only in managing exacerbations. However, clinicians are frightened by the risk of side effects, even repeating brief cycles during the course of a single year. Furthermore, the respondents seem not to perceive the recommendations from different multidisciplinary guidelines as consistent. For this reason, we agree on the need for clear guidelines on oral steroids, which could help support the use of a precision medicine approach, including indications for biological agents. In general, the authors agreed on the need for therapies that can reduce the use of systemic corticosteroids in patients with CRSwNP and that the new biologic agents may help address this unmet clinical need.

## Figures and Tables

**Table 1:** Aspects that should be pointed out during counselling when prescribing a topical corticosteroid to avoid side effects.

Options	Answers%
How to use the nasal spray	0.69%
How to position the device	58.62%
Position of the body	33.33%
Time of day to administer it	39.77%
When to do it with respect to nasal washing (before or after)	68.51%
Other	2.53%

**Table 2:** Strategies adopted by clinicians to reduce the risk of adverse events with local nasal corticosteroids.

Options	Answers%
Teach the patient how to use it correctly	49.89%
Discontinue the drug for at least 10 days per month	39.31%
Administration in alternate weeks	3.68%
Administration 15 days yes and 15 no	15.40%
Administration on alternate days	58.39%
Use of adjuvant drugs (hyaluronic acid, emollients, etc.)	49.89%
None of the above	3.91%
Other	1.61%

## Data Availability

All the data are available in the Supplementary Materials File.

## Supplementary Materials

The full questionnaire administered to the participants of the survey is available at https://www.mdpi.com/article/10.3390/jpm12060897/s1.
