# Supplementary material for: Survey on Use of Local and Systemic Corticosteroids in the Management of Chronic Rhinosinusitis with Nasal Polyps: Identification of Unmet Clinical Needs
Source: J Pers Med. 2022 May 29;12(6):897. doi: 10.3390/jpm12060897 (PMC9225345; doi:10.3390/jpm12060897)
Supplement: Supplementary file 1 [file jpm-12-00897-s001.zip › jpm-1732995-supplementary.pdf]

QUESTIONNAIRE ON USE OF TOPICAL AND SYSTEMIC CORTICOSTEROIDS IN THE MANAGEMENT OF CHRONIC RHINOSINUSITIS WITH NASAL POLYPS (CRSwNP)

USE OF INTRANASAL TOPICAL CORTICOSTEROIDS (OCS) IN PATIENTS WITH CRSWNP

Q1. WHICH LOCAL CORTICOSTEROID DO YOU MOST FREQUENTLY PRESCRIBE FOR TREATMENT OF CRSwNP IN YOUR PRACTICE?

Options	Answers%	Answers n.
Budesonide	28.97%	126
Fluticasone	8.51%	37
Mometasone furoate	79.31%	345
Tiamcinolone Acetonide	0.69%	3
Fluticasone in combination with azelastine	9.43%	41
Beclometasone dipropionate	10.80%	47
Other	1.15%	5
		Total: 435

Q2. WHAT METHOD OF ADMINISTRATION FOR LOCAL CORTICOSTEROIDS DO YOU MOST FREQUENTLY PRESCRIBE IN PATIENTS WITH CRSwNP?

Options	Answers%	Answers n.
Nasal spray	98.62%	430
Nasal drops	1.15%	5
Nasal washes (e.g. Rinowash)	16.97%	74
Aerosol	5.73%	25
Squeeze bottle (e.g. Rinoway/sinus rinse)	5.96%	26
Other	0.92%	4
		Total: 436

Q.3 IN YOUR PRACTICE, IN GENERAL, DO YOU ASSOCIATE LOCAL CORTICOSTEROIDS WITH NASAL WASHES OF PHYSIOLOGICAL SOLUTION?

Options	Answers%	Answers n.
Yes, always at the same time of the day	50.69%	221
Yes, always but at different times of the day	25.46%	111
Only in selected cases	18.12%	79
No, never	4.13%	18
Other	1.61%	7
		Total: 436

Q4. HOW LONG DO YOU RECOMMEND TREATMENT WITH A LOCAL CORTICOSTEROID IN PATIENTS WITH CRwNP AT THE FIRST EVALUATION BEFORE THE NEXT VISIT?

Options	Answers%	Answers n.
Short period of time (one month)	6.88%	30
Short period of time (two months)	13.30%	58
Short period of time (<3 months)	13.07%	57
Long period of time (>3 months)	41.28%	180
Long period of time (>6 months)	12.61%	55
Long period of time (12 months)	8.94%	39
Other	3.90%	17
		Total: 436

Q5. AT WHAT FREQUENCY DO YOU RECOMMEND A LOCAL CORTICOSTEROID IN PATIENTS WITH CRSwNP?

Options	Answers%	Answers n.
Every day	61.93%	270
Alternate days	1.38%	6
Alternate weeks	2.06%	9
15 days on and 15 days off	19.95%	87
Alternate months	4.82%	21
Other	9.86%	43
		Total: 436

Q6. IN YOUR EXPERIENCE, WHAT PERCENTAGE OF PATIENTS ADHERE TO INTRANASAL CORTICOSTEROID THERAPY AND LONG-TERM NASAL WASHINGS (>3 MONTHS)?

Options	Answers%	Answers n.
>90%	18.58%	81
70%	43.35%	189
50%	30.50%	133
30%	4.59%	20
<30%	2.52%	11
Other	0.46%	2
		Total: 436

Q7. DO YOU AGREE WITH THE STATEMENT "PROLONGED USE (> 3 MONTHS) OF INTRANASAL CORTICOSTEROIDS IS BURDENED BY MINIMAL LOCAL SIDE EFFECTS (EPISTAXIS) AND FEW/NO SYSTEMIC SIDE EFFECTS (E.G.: DIABETES, ALTERATIONS OF THE HYPOTHALAMUS-PITUITARY AXIS)"?

Options	Answers%	Answers n.
Fully agree	69.95%	305
Partially agree	20.87%	91
Partially disagree	5.05%	22
Fully disagree	4.59%	20
Other	4.13%	18
		Total: 436

Q8. WHEN PRESCRIBING A TOPICAL CORTICOSTEROID, WHICH OF THE FOLLOWING DO YOU PAY ATTENTION TO?

Options	Answers%	Answers n.
How to use the nasal spray	0.69%	3
How to position the device	58.62%	255
Position of the body	33.33%	145
Time of day to administer it	39.77%	173
When to do it with respect to nasal washing (before or after)	68.51%	298
Other	2.53%	11
		Total: 435

Q9. DO YOU AGREE WITH THE STATEMENT "IN CLINICAL PRACTICE, IT IS INDICATED TO MODULATE CORTICOSTEROID THERAPY BASED ON SEVERITY OF SYMPTOMS"? MODULATION MEANS BOTH DRUG DISCONTINUATION FOR SHORT PERIODS (EVERY OTHER DAY, 20 DAYS ON AND 10 DAYS OFF) AND MODULATING THE DAILY DOSE (TWICE PER DAY OR ONCE PER DAY)?

Options	Answers%	Answers n.
Fully agree	63.91%	278
Partially agree	28.51%	124
Partially disagree	5.06%	22
Fully disagree	63.91%	278
Other	2.53%	11
		Total: 435

Q10. IN YOUR CLINICAL PRACTICE, WHAT IS THE MOST COMMON EXTRANASAL OR SYSTEMIC SIDE EFFECT DURING PROLONGED USE (>3 MONTHS) OF LOCAL INTRANASAL CORTICOSTEROIDS?

**Options**	**Answers%**	**Answers n.**
Alterations in glucose metabolism (diabetes, carbohydrate intolerance)	4.60%	20
Alterations in bone metabolism (osteopenia, osteoporosis)	1.61%	7
Mood alterations	3.45%	15
Increased appetite	0.92%	4
Insomnia	5.98%	26
Hypertension	8.74%	38
Adrenal suppression	0.69%	3
No systemic effects	78.85%	343
Other	4.83%	21
		Total: 435

Q11. WHAT ADVERSE EFFECTS DID YOU MOST FREQUENTLY SEE AFTER PROLONGED USED OF LOCAL INTRANASAL CORTICOSTEROIDS?

**Options**	**Answers%**	**Answers n.**
Epistaxis	76.38%	333
Craniofacial pain and/or headache	2.06%	9
Hyposmia	1.38%	6
Hiccups	7.34%	32
Nasal dryness	70.41%	307
Increase in ocular pressure	5.96%	26
Cataract	0.46%	2
Nasal crusting	14.91%	65
Septal perforation	0.23%	1
Mucosal atrophy	10.78%	47
No local effects	4.82%	21
Other	1.15%	5
		Total: 436

Q12. WHICH OF THESE FACTORS DO YOU SEE AS A CONTRAINDICATION TO THE USE OF TOPICAL NASAL CORTICOSTEROIDS?

**Options**	**Answers%**	**Answers n.**
Risk of hemorrhage	44.19%	190
Hypertension	7.44%	32
Glaucoma	59.07%	254
Cataract	5.81%	25
Diabetes	10.93%	47
Immunosuppression	11.63%	50
Herpes simplex	29.07%	125
Other	5.58%	24
		Total: 430

Q13. WHICH OF THESE STRATEGIES MAY REDUCE THE RISK OF ADVERSE EVENTS WITH LOCAL NASAL CORTICOSTEROIDS?

**Options**	**Answers%**	**Answers n.**
Teach the patient how to use it correctly	49.89%	217
Discontinue the drug for at least 10 days per month	39.31%	171
Administration in alternate weeks	3.68%	16
Administration 15 days yes and 15 no	15.40%	67
Administration in alternate days	58.39%	254
Use of adjuvant drugs (hyaluronic acid, emollients etc.)	49.89%	217
None of the above	3.91%	17
Other	1.61%	7
		Total: 435

Q14. PRIOR TO SURGERY, DO YOU DISCONTINUE TOPICAL NASAL CORTICOSTEROID THERAPY?

**Options**	**Answers%**	**Answers n.**
No, I have the patient take it until the day of the surgery	71.66%	311
Yes, at least one week before	23.96%	104
Yes, one month before	2.76%	12
Yes, three months before	0.23%	1
Other	1.38%	6
		Total: 434

Q15. WHEN DO YOU THINK IT APPROPRIATE TO START INTRANASAL TOPICAL CORTICOSTEROID AFTER FESS?

Options	Answers%	Answers n.
In the immediate post-operative period after possible removal of nasal packing	7.37%	32
As soon as the mucosa in the entire nasal cavity has recovered.	29.03%	126
1 week after intervention	4.61%	20
15 days after intervention	15.90%	69
1 month	24.19%	105
2 months	3.92%	17
3 months	4.15%	18
6 months	0.69%	3
Only if recurrence occurs during follow-up	9.22%	40
Other	0.92%	4
		Total: 434

USE OF SYSTEMIC CORTICOSTEROIDS (OCS) IN TREATMENT OF CRSWNP

Q16. WHICH SYSTEMIC CORTISONE DO YOU MOST FREQUENTLY PRESCRIBE TO TREAT RECURRENCES?

Options	Answers%	Answers n.
Prednisone	45.73%	198
Prednisolone	10.16%	44
Methylprednisolone	19.63%	85
Deflazacort	38.80%	168
Hydrocortisone	1.15%	5
Betamethasone	11.55%	50
Triamcinolone	8.55%	37
Other	2.31%	10
		Total: 433

Q17. WHEN DO YOU THINK IT NECESSARY TO PRESCRIBE A SHORT COURSE OF ORAL STEROIDS IN THE MEDICAL TREATMENT OF CRSwNP?

Options	Answers%	Answers n.
Whenever there is a clinical exacerbation of symptoms, regardless of the number	20.28%	88
In case of exacerbation, trying not to exceed 2 cycles per year	41.01%	178
In case of exacerbation, trying not to exceed 3 cycles per year	13.36%	58
In case of exacerbation, trying not to exceed 4 cycles per year	3.00%	13
Once a month for 5 consecutive days each month	1.15%	5
Once a month for 7 consecutive days each month	19.12%	83
Other	2.07%	9
		Total: 434

Q18. IN THE CASE THAT IT IS NECESSARY TO PRESCRIBE A SHORT COURSE OF SYSTEMIC STEROIDS, WHAT DOSE DO YOU USUALLY PRESCRIBE TAKING PREDNISONE AS A REFERENCE?

**Options**	**Answers%**	**Answers n.**
50 mg per day regardless of weight	11.32%	49
25 mg per day regardless of weight	47.11%	204
10 mg per day regardless of weight	5.08%	22
5 mg per day regardless of weight	2.54%	11
I tailor the dose to the patient's weight	31.41%	136
Other	2.54%	11
		Total: 433

Q19. IF IT IS NECESSARY TO PRESCRIBE A SHORT COURSE OF SYSTEMIC STEROIDS IN A PATIENT WITH CRSwNP, FOR HOW MANY DAYS DO YOU ADMINISTER IT?

**Options**	**Answers%**	**Answers n.**
<5 days	3.23%	14
5-10 days	62.44%	271
10-15 days	27.42%	119
15-20 days	5.76%	25
20-25 days	0.23%	1
25-30 days	0.69%	3
Other	0.23%	1
		Total: 434

Q20. IN YOUR OPINION, WHICH OF THE FOLLOWING CAN BE USED TO DEFINE SEVERE UNCONTROLLED DISEASE?

**Options**	**Answers%**	**Answers n.**
At least one short course of systemic steroids per year in the last two years	4.14%	18
At least two short courses of systemic steroids in the last year	22.07%	96
At least three short courses of systemic cortisone in the last year	41.38%	180
At least four short courses of systemic cortisone in the last year	32.41%	141
		Total: 435

Q21. DO YOU AGREE WITH THE FOLLOWING STATEMENT? (IN MEDICAL TREATMENT OF CRSwNP IT IS PREFERABLE TO AVOID LONG-TERM LOW-DOSE ADMINISTRATION OF SYSTEMIC CORTICOSTEROIDS)

**Options**	**Answers%**	**Answers n.**
Fully agree	54.73%	237
Partially agree	31.87%	138
Partially disagree	10.39%	45
Fully disagree	3.00%	13
		Total: 433

Q22. DO YOU PRESCRIBE SYSTEMIC DEPOT STEROIDS (E.G. TRIAMCINOLONE ACETONIDE) IN YOUR CLINICAL PRACTICE TO TREAT CRSwNP?

**Options**	**Answers%**	**Answers n.**
Prefer not to prescribe them	35.88%	155
Never prescribed them	35.88%	155
In case of non-response to oral corticosteroids	12.04%	52
In management of simple forms/those not indicated for surgery	4.63%	20
For rapid control of symptoms	12.73%	55
For rapid recovery of olfaction	4.17%	18
Only in cases of non-response to a single dose of oral cortisone	9.72%	42
In the long-term management of CRSwNP	6.25%	27
		Total: 432

Q23. DO YOU AGREE WITH THE FOLLOWING STATEMENT? (THE INTERNATIONAL LITERATURE PROVIDES CLEAR GUIDELINES REGARDING THE POSOLOGY OF ORAL STEROIDS IN THE TREATMENT OF CRSwNP).

**Options**	**Answers%**	**Answers n.**
Fully agree	19.03%	82
Partially agree	56.84%	245
Partially disagree	17.40%	75
Fully disagree	6.73%	29
		Total: 431

Q24. DO YOU PRESCRIBE SYSTEMIC CORTICOSTEROID THERAPY PRIOR TO SURGERY? (E.G.: 5 DAYS BEFORE SURGERY)

Options	Answers%	Answers n.
No	29.79%	129
Yes always	24.71%	107
Yes, but based on severity of disease	42.03%	182
Only in cases of recurrence	1.85%	8
Other	1.62%	7
		Total: 433

Q25. IN THE EVENT OF COMORBIDITIES AT RISK FOR THE ADMINISTRATION OF SYSTEMIC CORTICOSTEROIDS (HYPERTENSION, GLAUCOMA, DIABETES, GASTRITIS, ETC.) HOW DO YOU MANAGE EXACERBATION OF SYMPTOMS IN CRSwNP?

Options	Answers%	Answers n.
Absolutely avoid prescribing systemic corticosteroids	14.62%	63
Proceed with long-term low-dose administration	7.66%	33
Use a systemic corticosteroid with low mineralocorticoid activity	17.87%	77
Proceed with the prescription with strict monitoring for adverse events	28.54%	123
Increase the dose of local corticosteroid therapy	11.14%	48
Use other medications such as antihistamine, antileukotriene agents, etc.	18.79%	81
Other	1.39%	6
		Total: 431

Q26. WHAT ADVERSE EVENTS HAVE YOU MOST FREQUENTLY OBSERVED WITH SYSTEMIC STEROIDS?

Options	Answers%	Answers n.
None	7.83%	34
Hyperglycemia	55.76%	242
Gastric reflux	29.49%	128
Increased appetite	12.67%	55
Mood changes	21.66%	94
Insomnia	50.00%	217
Anxiety	23.27%	101
Cataract	1.38%	6
Glaucoma	10.83%	47
Hypertension	57.60%	250
Diabetes	23.04%	100
Increased susceptibility to infections	3.69%	16
Adrenal suppression	2.30%	10
Osteoporosis	6.68%	29
Other	1.15%	5
		Total: 434

Q27. HOW DO YOU THINK IT IS APPROPRIATE TO EVALUATE SYSTEMIC CORTISONE USE OF PATIENTS WITH CRSwNP?

Options	Answers%	Answers n.
Consider the number of cycles that the patient has undergone in the last year	62.59%	271
Consider the total number of days in which the patient has taken a systemic cortisone in the last year	16.86%	73
Consider the total dose that the patient has taken in the last year	17.78%	77
Consider the number of cycles the patient has taken in the last six months	12.70%	55
Consider the total number of days in which the patient has taken a systemic cortisone in the last six months	6.47%	28
Consider the total dose that the patient has taken in the last six months of the last year	6.70%	29
		Total: 433

Q28. WHAT ANNUAL DOSAGE OF SYSTEMIC CORTICOSTEROIDS DO YOU CONSIDER DANGEROUS DUE TO THE HIGH RISK OF ADVERSE EVENTS CONSIDERING TOTAL DAYS OF USE?

**Options**	**Answers%**	**Answers n.**
More than 3 weeks in a year	11.34%	49
More than 4 weeks in a year	34.95%	151
More than 5 weeks in a year	10.88%	47
More than 6weeks in a year	22.92%	99
More than 7 weeks in a year	3.01%	13
More than 8 weeks in a year	16.90%	73
		Total: 432

Q29. WHAT TOTAL ANNUAL DOSE OF SYSTEMIC CORTICOSTEROIDS DO YOU CONSIDER DANGEROUS DUE TO THE HIGH RISK OF ADVERSE EVENTS (TOTAL AMOUNT CORTISONE IN MG TAKEN IN THE LAST YEAR)?

**Options**	**Answers%**	**Answers n.**
200 mg of prednisone	3.97%	17
400 mg of prednisone	11.21%	48
800 mg of prednisone	18.93%	81
1000 mg of prednisone	23.13%	99
1200 mg of prednisone	7.94%	34
1400 mg of prednisone	7.24%	31
1600 mg of prednisone	8.41%	36
1800 mg of prednisone	3.97%	17
2000 mg of prednisone	11.21%	48
Other	3.97%	17
		Total: 428

Q30. WHICH OF THE FOLLOWING DO YOU MONITOR FOR THE ONSET OF ADVERSE EVENTS RELATED TO ADMINISTRATION OF SYSTEMIC CORTICOSTEROID THERAPY AND/OR RECOMMEND TO THE PATIENT:

**Options**	**Answers%**	**Answers n.**
Blood pressure	90.99%	394
Glycemia	87.53%	379
Blood tests for levels of cortisol and ACTH	10.85%	47
Recommend dosage of 24-hour urinary cortisol	2.31%	10
Refer to colleagues (endocrinologist, etc.)	18.01%	78
Other …	3.00%	13
		Total: 433

Q31. HAVE YOU EVER DISCONTINUED A SYSTEMIC CORTICOSTEROID IN YOUR PATIENTS? IF YES, FOR WHICH ADVERSE EVENTS?

Options	Answers%	Answers n.
Never	23.33%	101
Discontinued for osteoporosis	4.39%	19
Discontinued for hyperglycemia and/or diabetes mellitus	43.19%	187
Suspended for hypertensive peaks	54.04%	234
Discontinued for dyslipidemia, weight gain	2.08%	9
Discontinued for gastritis and/or peptic ulcer	21.02%	91
Discontinued for cataract, glaucoma	11.78%	51
Discontinued for anxiety, depression, sleep disturbance	27.25%	118
Discontinued for other reasons	2.54%	11
Other...	0.92%	4
		Total: 433

Q32. BASED ON YOUR CLINICAL EXPERIENCE, WHAT PERCENTAGE OF PATIENTS ADHERE TO THE ORAL SYSTEMIC CORTICOSTEROID THERAPY PRESCRIBED?

Options	Answers%	Answers n.
90 %	36.43%	157
70%	40.60%	175
50%	16.94%	73
30%	3.25%	14
<30%	1.16%	5
Other	1.62%	7
		Total: 431

Q33. IN THE EVENT THAT AN OLFACTORY DISORDER IS THE PREVALENT SYMPTOM ASSOCIATED WITH CRSwNP, DO YOU RECOMMEND A COURSE OF ORAL STEROID THERAPY?

Options	Answers%	Answers n.
Yes, always	27.55%	119
No, never	6.25%	27
Yes, but if the patient has already completed a course of local corticosteroid therapy	49.07%	212
Yes, but only if the patient refers anosmia	7.64%	33
Yes, even if the patient refers hyposmia	7.64%	33
Other	1.85%	8
		Total: 432

Q34. IF A PATIENT WITH CRSwNP COMPLAINS MAINLY OF OLFACTORY DISTURBANCES AND ORAL STEROID THERAPY IS INDICATED, FOR HOW MANY DAYS DO YOU PRESCRIBE IT?

Options	Answers%	Answers n.
Short cycle of 7 days at full dosage per kg	49.31%	213
Short course of 7-day low-dose therapy	13.19%	57
Short cycle of 15 days at full dosage per kg	22.22%	96
Short 15-day low dose cycle	8.56%	37
3-week full-dose cycle per kg	2.08%	9
3-week cycle at a reduced dose	0.69%	3
One month of full-dose therapy per kg	0.46%	2
One month of reduced dose therapy	0.46%	2
Long-term low-dose cortisone	0.46%	2
Depot corticosteroid	0.93%	4
Other	1.62%	7
		Total: 432

Q35. IF A PATIENT WITH CRSwNP COMPLAINS MAINLY OF OLFACTORY DISTURBANCES AND ORAL STEROID THERAPY IS INDICATED, WHAT AGENT DO YOU USE MOST FREQUENTLY?

Options	Answers%	Answers n.
Prednisone	45.14%	195
Prednisolone	7.87%	34
Deflazacort	29.17%	126
Methyl prednisolone	10.42%	45
Betamethasone	5.56%	24
Hydrocortisone	0.23%	1
Other	1.62%	7
		Total: 432

Q36. IN YOUR OPINION, WHAT IS THE EFFECTIVENESS OF OCS IN CLINICAL PRACTICE IN RESTORING OLFACTION IN CRSwNP?

Options	Answers%	Answers n.
Long-lasting improvement of smell	15.38%	66
Only temporary improvement of smell	69.70%	299
Insufficient recovery of smell in general	9.79%	42
Insufficient recovery of smell in patients with history of multiple surgical interventions	3.73%	16
Other	1.40%	6
		Total: 429

Q37. IN YOUR EXPERIENCE, IF A PATIENT HAS OLFACTORY RECOVERY WITH PREOPERATIVE OCS IS THIS PREDICTIVE OF GOOD OLFACTORY RECOVERY AFTER FESS?

Options	Answers%	Answers n.
No, never	2.30%	10
No, because I don't think it is predictive of olfactory recovery	16.36%	71
I do not usually prescribe pre-operative cortisone	14.29%	62
Yes, because it means that the patient has a good olfactory reserve	34.10%	148
Yes, although it doesn't ensure long-lasting benefit	32.95%	143
		Total: 434

Q38. DO YOU AGREE WITH THE FOLLOWING STATEMENT: "IN THE EVALUATION OF CUMULATIVE ANNUAL DOSE OF OCS, IT SHOULD BE TAKEN INTO ACCOUNT THE OCS ADMINISTERED FOR ASTHMA"?

Options	Answers%	Answers n.
Fully agree	68.28%	297
Partially agree	25.29%	110
Partially disagree	4.37%	19
Disagree	2.07%	9
Only if I want to prescribe an oral steroid for sinusitis	0.00%	0
Never, since the steroid is prescribed for asthma	0.00%	0
		Total: 435
